# Relationship Between Attention Bias and Psychological Index in Individuals With Chronic Low Back Pain: A Preliminary Event-Related Potential Study

**DOI:** 10.3389/fnhum.2020.561726

**Published:** 2020-10-26

**Authors:** Takayuki Tabira, Michio Maruta, Ko Matsudaira, Takashi Matsuo, Takashi Hasegawa, Akira Sagari, Gwanghee Han, Hiroki Takahashi, Jun Tayama

**Affiliations:** ^1^Department of Clinical Neuropsychiatry, Graduate School of Health Science, Kagoshima University, Kagoshima, Japan; ^2^Doctoral Program of Clinical Neuropsychiatry, Graduate School of Health Science, Kagoshima University, Kagoshima, Japan; ^3^Department of Medical Research and Management for Musculoskeletal Pain, 22nd Century Medical and Research Center, Faculty of Medicine, The University of Tokyo-Hospital, Tokyo, Japan; ^4^Division of Occupational Therapy, Department of Rehabilitation, Kumamoto Health Science University, Kumamoto, Japan; ^5^Unit of Medical Science, Graduate School of Biomedical Sciences, Nagasaki University, Nagasaki, Japan; ^6^Department of Occupational Therapy, School of Health Sciences, Faculty of Medicine, Shinshu University, Nagano, Japan; ^7^Department of Neuropsychiatry, Kumamoto University Hospital, Kumamoto, Japan; ^8^Department of Rehabilitation Center, Nagasaki University Hospital, Nagasaki, Japan; ^9^Faculty of Human Sciences, Waseda University, Saitama, Japan

**Keywords:** attention bias modification, chronic low back pain, event-related potentials (ERP), psychological index, individuals

## Abstract

**Introduction**: Attention bias towards pain-related information exists in patients with chronic pain, and recently, attention bias modification (ABM) training has been administered to patients with chronic pain. In this study, we conducted an attention bias modification task in conjunction with event-related potential measurements for individuals with chronic low back pain (LBP) and investigated the relationship between attention bias and psychological assessment.

**Methods**: Eleven women and two men with chronic LBP participated in the study.

**Results**: The Japanese version of the STarT Back Screening Tool (J-SBST) total score was significantly correlated with the N1 amplitude of Cz. The J-SBST psychological score was significantly correlated with the N1 amplitude of Cz and with reaction time (RT). The Japanese version of the Pain Catastrophizing Scale (PCS) and Japanese version of the Beck Depression Inventory-Second Edition (BDI-II) scores were significantly correlated with the P2 amplitude at Fz (only PCS), Cz, and Pz.

**Conclusions**: Our findings suggest that J-SBST, which provides a comprehensive evaluation of psychological factors, PCN with measuring of catastrophizing in the context of actual or anticipated pain, and BDI-II, can likely help identify chronic LBP patients with attention bias. For chronic LBP patients who are classified according to J-SBST or PCN pain-related outcome improvement with ABM training can be expected.

## Introduction

The role of attention processing in chronic pain is important (Pincus and Morley, 2001), and many studies have investigated the existence of attention bias towards pain-related stimuli. A meta-analysis investigating attention bias to pain-related information indicates that attention bias towards pain-related words or pictures exists in people with chronic pain (Schoth et al., 2012; Crombez et al., 2013). Attention bias is generally divided into avoidance and hypervigilance from the direction of the bias of attention, and Herbert et al. (2014) reported that pain hypervigilance is associated with pain intensity and clinical disability, as well as enhanced pain sensitivity. Attention bias to pain can lead to an increased disability, enhanced pain catastrophizing, and avoidance of activities. Psychological factors, including fear-avoidance beliefs or somatizing tendency, had a significant association with chronic low back pain (LBP) among care workers (Yoshimoto et al., 2019). The Subgrouping for Targeted Treatment Back (STarT Back) Screening Tool (SBST) to assess and stratify patients with LBP according to the risk of LBP chronicity as psychological factors has been globally used and indicated that stratification of risk groups by the Japanese version of the SBST (J-SBST) may help predict prognosis of LBP (Matsudaira et al., 2016, 2017).

In the literature related to anxiety, attention bias towards threat has been indicated (Bar-Haim et al., 2007; Bar-Haim, 2010). Attention bias modification (ABM) is a recently developed psychological intervention to modify attention bias towards negative stimuli for such anxiety disorders (Bar-Haim et al., 2007; Hakamata et al., 2010). Numerous reports confirm the effectiveness of ABM, particularly effective for reducing threat bias and anxiety symptoms in people with generalized anxiety and social phobia (Amir et al., 2009a,b; Schmidt et al., 2009). The dot-probe task is a widely used method for assessing attentional bias (MacLeod et al., 1986). In the dot-probe task, a randomized pair of stimuli, one which is neutral and the other, a threat-perception negative emotion, is presented on the upper and lower portions of a screen, respectively; the neutral stimulus is chosen over the stimulus that causes negative emotion. Repeating these tasks provides a way to desensitize negative emotions. MacLeod et al. (2002) developed a computerized task to train participants to attend away from a negative stimulus. ABM has been applied not only to anxiety (Hakamata et al., 2010; Tayama et al., 2018) but is also used for smokers (Attwood et al., 2008) and alcoholic patients (Schoenmakers et al., 2010), and the effects have been reported.

Recently, ABM training has also been administered to chronic pain patients (Dehghani et al., 2004; Sharpe et al., 2012; Schoth et al., 2013; Heathcote et al., 2017). For example, in a randomized controlled trial for 34 chronic pain patients, the ABM training group showed a significant reduction in pain-related outcomes, such as anxiety sensitivity and functional disability than did the placebo group (Sharpe et al., 2012). However, a randomized controlled trial for 66 adolescents with chronic pain reported that there was no evidence that ABM changed attentional bias or that pain-related outcomes differed between the ABM and placebo or no-training groups (Heathcote et al., 2017). In patients with chronic pain, the effectiveness of ABM training is not well established and further studies are required.

Reaction time (RT) is usually used as an index of attention bias in the dot-probe task, however, poor internal reliability is indicated (Kappenman et al., 2014). Therefore, in addition to the RT index of attention bias, recent studies measure event-related potentials (ERPs) of the electroencephalogram (EEG) in conjunction with the dot-probe task (Holmes et al., 2009; Kappenman et al., 2015; Gibb et al., 2016). The ERPs exhibit superior temporal resolution and can provide a more direct measure of attention allocation in attention bias in conjunction with the dot-probe task. In ERPs study of patients with anxiety, P140 amplitude was increased using a visual dot-probe task (Rossignol et al., 2013), and N200 amplitude was increased using emotion-word Stroop task (Sass et al., 2014), and initial shift in attention to threat stimuli has been identified. The parietal P100 component is an early visual ERP component whose amplitude and latency are affected by the neural processing of facial expressions (Kolassa et al., 2006). N1 reflects feature detection and sensory attention capture based on the salience of the stimulus from two visual-detection experiments (Wascher et al., 2009), and maybe attributed to increased efforts to divert attention away from visual threat stimuli (Dennis and Chen, 2007). The generator mechanisms are not fully understood, it is classically known that sensory regions are one of the generators of N1 (Picton et al., 1976). P2 has been associated with the processing of emotion in faces (Carretié et al., 2001), and was a neural response that is sensitive to threat-related stimuli using dot-probe task (O’Toole and Dennis, 2012). N2 component reflects attention control and inhibition mechanisms (Falkenstein et al., 1999; Flostein and Van Perren, 2008), and maybe attributed to increased efforts to divert attention away from visual threat stimuli (Dennis and Chen, 2007). P3 has been associated with the strategic orienting of attention (Friedman et al., 2001; Fichtenholtz et al., 2007). In the attention bias task in patients with anxiety, the discussion is divided such as slight appear (Eldar and Bar-Haim, 2010; O’Toole and Dennis, 2012) and not appear (Dennis-Tiwary et al., 2016; Tayama et al., 2018), and early components are receiving more attention. However, only a few studies on attention bias in patients with chronic pain have used ERP measurements.

In this study, we have conducted the ABM task in conjunction with ERP measurements for individuals with chronic low back pain (LBP), which has a high prevalence in Japan (Nakamura et al., 2011). This study aimed to clarify the relationship between attention bias and psychological assessments of individuals with chronic LBP, we examined the attentional component of the ERPs as well as the RT in the ABM to determine whether patients with chronic LBP who have higher socio-psychological factors such as fear-avoidance, catastrophizing and depression show more attentional bias to threat stimuli, therefore, this study can provide psychophysiological insight into how the psychological domains and its severity in individuals with chronic LBP relate to attention bias using ERP as well as RT. We contribute to the development of ABM training, occupational therapy, and management in individuals with LBP.

## Materials and Methods

### Participants

A total of 11 women and two men with chronic LBP were recruited from the local community (mean age: 70.3 ± 8.3 years). Participants met the following inclusion criteria: (1) a minimum of a 6-month history of pain; (2) absence of neurological or psychiatric disorders; (3) absence of other chronic disorders. All participants had a normal or corrected-to-normal vision. The study was approved by the research ethics committee of Nishikyushu University and was conducted following the Declaration of Helsinki.

### Attention Bias Modification Task

We used a personal computer (AT992; EPSON, Nagano, Japan), a 19-inch monitor (Pro-Lite E1980SD; Iiyama, Tokyo, Japan), and an image controller (MTS0410; Medical Try System, Tokyo, Japan) for the ABM task. The distance between the participant and the center of the monitor display was about 65 cm. We used facial images of eight different people from The Japanese Female Facial Expression database as visual stimuli. Neutral and threat (angry or fear) facial expression images were used for the task.

**Figure 1:**
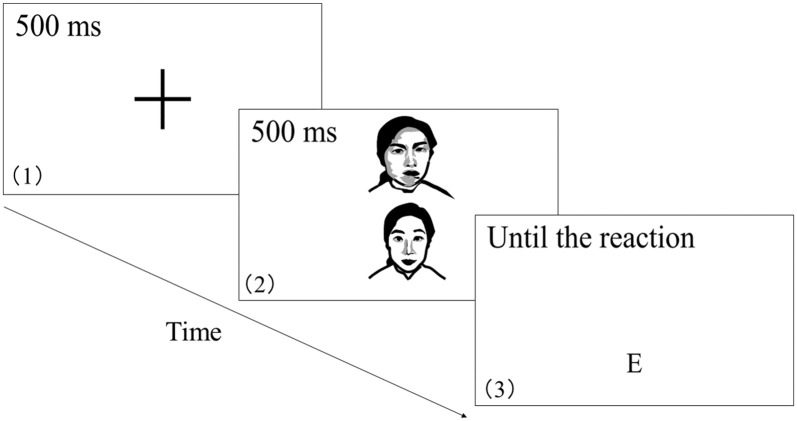
The procedure of attention bias modification (ABM) task. The image presentation sequence and duration were as follows: (1) fixation cross was presented for 500 ms; (2) target image pair was presented for 500 ms; (3) symbol was presented at the bottom of the screen until the participant pressed the button. Participants were required to indicate the position of the neutral face as rapidly and accurately as possible by pressing one of two buttons on a button box.

On each trial, a randomized pair of neutral and threat facial expressions were presented against a white background on the upper and lower portions of the screen, respectively. The ABM task consisted of three blocks. Following a 500-ms presentation of a fixation cross at the center of the screen, the target image pair was presented for 500 ms. Following the removal of the images, a symbol (“E”) was presented at the bottom of the screen until the participant pressed the button (Figure 1). Participants were required to indicate the position of the neutral face as rapidly and accurately as possible by pressing one of two buttons on a button box using the middle or index finger of the dominant hand. RT was measured starting at probe presentation. Trial with RTs that were <200 ms or >1,000 ms and those with incorrect answers were excluded from the analysis (Dehghani et al., 2004). Each participant performed 128 trials.

### EEG Recordings and Analysis

We used the Neuropack X1 MEB-2300 series electromyogram measuring system (Nihon Kohden Corporation, Tokyo, Japan) for EEG measurements and the EPLYZER2 (Kissei Comtec, Matsumoto, Japan) for waveform analysis. The EEGs were recorded with Ag/AgCl disk electrodes placed at the Fz, Cz, and Pz positions (Mühlberger et al., 2009; Tayama et al., 2018) according to the International 10-20 system. Each scalp electrode was referenced to linked earlobes. The ground electrode was placed at the Fpz position. To eliminate eye movements or blinks exceeding 100 μV, electrooculograms were also recorded. Also, subject muscle movements were monitored and recorded on video. Electrode impedance was maintained below 5 kΩ. The EEG was digitized at a sampling rate of 1,000 Hz. EEG data in the range of 200 ms pre-stimulus to 600 ms post-stimulus were epoched. The N1 and N2 peaks were measured as the voltage at the most negative peak in the latency window of 100–150 ms and 150–300 ms after stimulus onset at all electrode positions. The P1, P2, and P3 peaks were at the most positive peak in the latency window of 50–100 ms, 100–200 ms, and 250–500 ms at all electrode positions. The final ERP waveforms were obtained by removing electrooculograms and muscle movements from the only waveforms of correct in the ABM task.

### Psychological Measurements

#### Japan Low Back Pain Evaluation Questionnaire (JLEQ)

The JLEQ is a 30-item, self-administered questionnaire including seven questions on LBP status in the previous few days (items 1–7), 17 questions on problems with activities of daily living due to LBP (items 8–24), and six questions on general health and psychological status in the previous month (items 25–30). Each of the questions was scored on a 5-point scale. The JLEQ scores provide a measure of the level of impairment in activities of daily living of patients with chronic LBP and have shown adequate validity and reliability (Shirado et al., 2007).

#### Japanese Version of the Fear Avoidance-Beliefs Questionnaire (FABQ)

The FABQ is a 16-item measure of fear-avoidance beliefs in patients with LBP (Waddell et al., 1993). Items 2–5 evaluate fear-avoidance beliefs about physical activity, and items 6, 7, 9–12, and 15 evaluate fear-avoidance beliefs about work. Each question is scored on a scale of 0–6. We evaluated fear-avoidance beliefs about physical activity. Good psychometric properties have been reported in Japanese workers with LBP (Matsudaira et al., 2014).

#### Japanese Version of the STarT Back Screening Tool (J-SBST)

The STarT Back has been widely used to stratify patients with LBP according to the risk for chronicity. The STarT Back consists of nine items. Items 1–4 evaluate physical factors, and items 5–9 assess psychosocial factors, related to LBP (Hill et al., 2008). Response options for items 1–8 are “disagree” (0 points) or “agree” (1 point). Responses to item 9 are on a scale of 1–5: “not at all,” “slightly,” “moderately,” “very much,” or “extremely.” The first three options (“not at all,” “slightly,” and “moderately”) are scored as 0, and the remaining two options (“very much” and “extremely”) are scored as 1. Good psychometric properties and validity have also been reported for the Japanese version of the STarT Back (J-SBST; Matsudaira et al., 2016).

#### Japanese Version of the Pain Catastrophizing Scale (PCS)

The PCS is a 13-item, self-administered questionnaire to measure pain catastrophizing and has shown high levels of reliability and validity (Sullivan et al., 1995). Each question is scored on a scale of 0–4. The total scores range from 0 to 52. Adequate reliability and validity have been also reported for the Japanese version (Matsuoka and Sakano, 2007).

#### Japanese Version of the Beck Depression Inventory-Second Edition (BDI-II)

The BDI-II, a widely-used, self-reporting instrument for measuring the severity of depression, consists of a 21-item questionnaire. Each question is scored on a scale of 0–3. The total scores range from 0 to 63. The Japanese version of the BDI-II has also been reported to exhibit adequate validity and reliability (Kojima et al., 2002).

### Statistical Analysis

Statistical calculations were carried out using IBM SPSS Statistics version 24.0 (IBM Corp., Armonk, NY, USA). The analysis of correlation was performed after checking data with a normal distribution using the Shapiro-Wilk test. If the normal distribution was confirmed, Pearson’s correlation was calculated. If non-parametric data were found, Spearman’s correlation was analyzed. We performed Pearson’s correlation analysis to determine whether the N1 amplitude, P2 amplitude, and RT are related to the psychological index. The analysis was performed on all data obtained from 13 participants. *P* < 0.05 was statistically significant.

## Results

### Latencies and Amplitudes of Each Component in ERP and RT in the ABM Task

The mean RT in the ABM task was 446.4 ± 137.5 ms. The number of correct in ABM tasks was more 99/128 in each participant, and the correct rate was 86.2 ± 7.4%. A total of 9.3 ± 5.5 contaminations of electrooculograms and muscle movements were removed from the ERP waveform of 110.3 ± 9.5 (86.2 ± 7.4%) correct answers in the ABM task. Finally, 101.0 ± 11.8 ERP waveforms were obtained.

**Figure 2:**
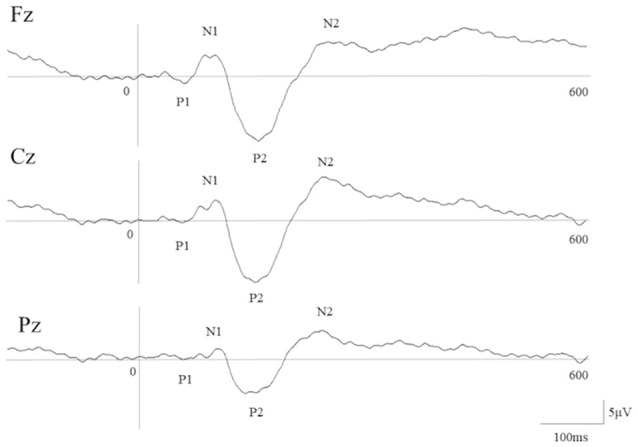
Grand-average of event-related potentials (ERPs) waveform in attention bias modification (ABM) task. This grand-average of ERP waveform is from 200 ms before to 600 ms after stimulation in the ABM task.

The grand-average of webform in ERP at Fz, Cz, and Pz were showed in Figure 2, and latencies and amplitudes of each component in ERPs were showed in Table 1. N1 and P2 were all detected, but P1 was detected in only six to eight participants (Fz;6, Cz;8, Pz;8), N2 was detected in only six to eight participants (Fz;8, Cz;8, Pz;6), P3 was detected in only one to two participants (Fz;1, Cz;1, Pz;2).

**Table 1:** Psychological index and pain-related score in each participant.

	JLEQ			FABQ	J-SBST		PCS	BDI-II
	Total score	LBP status score	Psychological score		Total score	Psychological score		
1	5	2	0	4	0	0	0	5
2	32	10	5	4	2	1	10	5
3	18	4	1	3	3	1	25	5
4	34	12	4	30	4	2	18	5
5	13	4	0	15	2	1	8	0
6	15	8	2	15	0	0	12	1
7	12	2	4	6	5	4	9	0
8	16	4	3	14	0	0	4	0
9	16	6	3	21	4	3	26	5
10	47	9	7	15	5	7	28	13
11	16	5	1	24	2	1	35	19
12	33	8	6	18	7	6	29	13
13	53	17	9	22	6	2	14	11
Average	21.9 ± 11.3	6.2 ± 3.0	3.4 ± 2.4	13.0 ± 8.7	3.1 ± 2.3	1.6 ± 1.5	16.6 ± 10.5	5.9 ± 5.8

*The average is presented as mean ± SD. JLEQ, Japan Low Back Pain Evaluation Questionnaire; FABQ, Japanese version of the Fear Avoidance-Beliefs Questionnaire; J-SBST, Japanese version of the Keele STarT Back screening tool; PCS, Japanese version of the Pain Catastrophizing Scale; BDI-II, Japanese version of the Beck Depression Inventor-Second Edition; LBP, Low back pain*.

### Psychological and Pain-Related Assessment Score

Table 2 shows the psychological index score of each participant.

**Table 2:** Latencies and amplitudes of each component in event-related potentials (ERPs).

	P1	N1	P2	N2
Latency (ms)				
Fz	73.3 ± 8.9	108.7 ± 15.7	161.8 ± 18.9	233.14 ± 15.1
Cz	76.2 ± 14.1	115.4 ± 19.4	161.7 ± 20.6	232.7 ± 15.2
Pz	76.7 ± 8.0	117.8 ± 17.9	160.8 ± 30.72	211.3 ± 7.9
Amplitude (μV)				
Fz	5.1 ± 5.6	−5.3 ± 7.2	14.5 ± 11.6	−7.4 ± 5.6
Cz	1.6 ± 3.3	−4.2 ± 4.3	15.7 ± 11.8	−7.3 ± 6.6
Pz	2.8 ± 6.2	−3.4 ± 3.2	8.3 ± 6.9	−5.8 ± 5.5

*The average is presented as mean ± SD. N1 and P2 were all detected, but P1 was detected in only 6–8 participants (Fz;6, Cz;8, Pz;8), N2 was detected in only 6–8 participants (Fz;8, Cz;8, Pz;6), P3 was detected in only 1–2 participants (Fz;1, Cz;1, Pz;2)*.

### Association Between Psychological Index Score and N1 Amplitude, P2 Amplitude, and RT

The N1 amplitudes of Cz showed a significant negative correlation with the STarT Back total scores (*r* = −0.646, *p* = 0.017), STarT Back psychological scores (*r* = −0.662, *p* = 0.014). The P2 amplitudes of Fz, Cz and Pz showed a significant negative correlation with the PCS scores (Fz; *r* = −0.634, *p* = 0.020, Cz; *r* = −0.705, *p* = 0.007, Pz; *r* = −0.615, *p* = 0.25) and BDI-II score (Cz; *r* = −0.743, *p* = 0.004, Pz; *r* = −0.604, *p* = 0.029). There was no significant correlation between theN1 amplitudes of Fz and Pz with any of the psychological indexes. RT showed a significant positive correlation with the STarT Back psychological scores (*r* = −0.605, *p* = 0.029). Table 3 shows the correlation coefficient between each psychological index and the N1 and P2 amplitudes, and RT.

**Table 3:** Correlation coefficient between each psychological index and the N1, P2 amplitudes and RT.

		JLEQ		FABQ	J-SBST		PCS	BDI-II
		Total score	Psychological score		Total score	Psychological score		
N1	Fz	0.312	0.234	−0.311	0.004	−0.066	0.449	0.196
	Cz	−0.083	−0.160	−0.271	−0.646*	−0.662*	−0.035	0.137
	Pz	0.204	−0.166	−0.165	−0.288	−0.380	0.500	−0.501
P2	Fz	−0.324	−0.487	−0.413	−0.454	−0.587	−0.634*	−0.545
	Cz	−0.426	−0.382	−0.477	−0.215	−0.121	−0.705**	−0.743**
	Pz	−0.442	−0.372	−0.340	−0.101	0.029	−0.615**	−0.604*
RT		−0.563	0.084	−0.133	0.322	0.605*	−0.019	−0.177

Pearson’s correlation coefficient, *p < 0.05. **p < 0.01. JLEQ, Japan Low Back Pain Evaluation Questionnaire; FABQ, Japanese version of the Fear Avoidance-Beliefs Questionnaire; J-SBST, Japanese version of the STarT Back screening tool; PCS, Japanese version of the Pain Catastrophizing Scale; BDI-II, Japanese version of the Beck Depression Inventor-Second Edition; RT, Reaction time.

## Discussion

In this study, attention bias measurement using the ABM task was performed for individuals with chronic LBP, and its relevance to the psychological index was investigated. Our results showed that higher J-SBST total and psychological scores were associated with larger N1 amplitudes of Cz, and higher PCS was associated with larger P2 amplitudes of Fz, Cz, and Pz. Higher BDI-II scores were associated with larger P2 amplitudes of Cz and Pz. Also, it was observed that longer RTs corresponded to the higher Psychological score of J-SBST.

ERPs can enable the investigation of responses of individuals related to internal and external events (Fonaryova Key et al., 2005), and the visual cognitive process is said to consist of an early automatic stage representing exogenous aspects and the late strategic stage representing endogenous aspects (Luck, 2014). The early components of ERPs, P1, N1, and P2, are exogenous components caused by external events, and the late components, N2 and P3, are endogenous components caused by internal events. The N1 and P2 components, which was associated with psychological indicators in the current study reflects exogenous automatic attention, and it is noted to be related to early emotion processing for N1 amplitude (Keil et al., 2001; Foti et al., 2009; Gable and Harmon-Jones, 2012), recognition processes for P2 amplitude (Halit et al., 2000). In an ERP study investigating emotional processing in social anxiety, the N1 amplitude to facial stimuli increased in the high social anxiety (HSA) group as compared with that in the low social anxiety group, which means that the HSA group showed an early attentional bias to facial expressions (Felmingham et al., 2016). The ERP study investigating attentional bias in obsessive-compulsive disorder (OCD) reported that the N1 and P2 amplitudes to OCD-related expression stimuli increased in the OCD group as compared with the healthy control group (Zhang et al., 2017). Since people with chronic pain also exhibit an attentional bias towards pain-related words or pictures (Schoth et al., 2012; Crombez et al., 2013), the N1 and P2 amplitudes to threat-related facial expression is considered to increase in people with chronic pain. Also, previous studies suggested that stimuli with negative emotionality elicited increased P2 amplitudes relative to a stimulus with positive emotionality (Carretié et al., 2001; Huang and Luo, 2006). Accordingly, participants with higher attention bias in this study should exhibit increased N1 and P2 amplitudes. In attention-bias measurement using the dot-probe task, the differences in RT to threat and neutral stimuli indicate attention bias (MacLeod et al., 1986), and people with attention bias toward negative information respond rapidly to a threat stimulus and the RT to a neutral stimulus is longer. Therefore, it can be interpreted that participants with longer RT in the current study exhibited an attention bias towards the threat stimuli.

In this study, participants with a higher total score of J-SBST showed increased N1 amplitudes of Cz and longer RTs. Also, participants with a higher psychological score of J-SBST showed increased N1 amplitudes of Cz. Furthermore, participants with a higher score of PCS and BDI-II showed increased P2 amplitudes of Cz and Pz. Our findings suggested that individuals with chronic LBP with high STarT Back or PCS or BDI-II had attention bias towards the threat stimulus.

In the Cz, N1 amplitude, which reflects feature detection and sensory attention capture was associated with J-SBST, which measures risk factors (especially, psychological factors) in individuals with chronic LBP. This suggests that higher psychological factors specific to individuals with chronic LBP may have generated sensory attention to threat stimuli, and N1 may have been enhanced by efforts to avoid threat stimuli. The P2 was also associated with PCS regardless of location. P2 has been associated with the processing of emotion in faces (Carretié et al., 2001) and attention disengagement (Bar-Haim et al., 2005), suggesting that P2 was more sensitive to discrimination of facial expression with the higher level of catastrophizing in chronic LBP, and P2 amplitudes may have been associated. The inclusion of Fz may be related to only discrimination of facial expression in near the parietal (O’Toole and Dennis, 2012), but also the prefrontal cortex, which contributes to cognition and emotion due to chronic pain (Price, 2000; Apkarian et al., 2004). In the relation between P2 and BDI-II, chronic pain patients have a higher incidence of depression (Sheng et al., 2017; Zis et al., 2017), and have attention bias toward the negative expressions (Kaiser et al., 2018), it may be a mechanism similar to PCS. Furthermore, the significant association between RT and the psychological score of J-SBST is consistent with previous studies on ABM in patients with chronic LBP (Dehghani et al., 2004; Sharpe et al., 2012; Schoth et al., 2013; Heathcote et al., 2017). It was suggested that individuals with more negative psychological states resulting from chronicity of LBP were more likely to pay attention to the threat stimuli, and took longer to select neutral stimuli.

STarT Back Screening Tool was originally developed as a screening tool to identify prognostic indicators of LBP to support primary care clinical decision-making in the UK and is widely used to stratify patients with LBP according to the risk for chronicity (Hill et al., 2008). STarT Back Screening Tool is an assessment tool that includes five carefully selected items, which are psychosocial risk factors. The Pain Catastrophizing Scale is a 13-item self-report measure of catastrophizing in the context of actual or anticipated pain (Sullivan et al., 1995). Attention bias is reported to be related to psychological factors such as anxiety (Bar-Haim et al., 2007), fear-avoidance (Hughes et al., 2017), catastrophizing (Michael and Burns, 2004; Heathcote et al., 2015) with negative mental set brought to bear on actual or anticipated pain, and thus, present results suggested that chronic LBP patients with attention bias towards the threat stimulus had a various influence on psychosocial aspects.

Also, Hill et al. (2011) administered treatment based on the results with the STarT Back Screening Tool and reported that the outcome with the cognitive-behavioral therapy (CBT)-added protocol was better for the high-risk group, for which psychological factors are considered to be strongly involved. Chronic pain is particularly susceptible to cognitive and psychological aspects, and in recent years several effects of CBT on chronic pain have been reported (Hoffman et al., 2007; Williams et al., 2012; Knoerl et al., 2016). CBT is also recommended for social anxiety disorder (SAD; Pilling et al., 2013) and meta-analyses have reported the effect of CBT on SAD (Mayo-Wilson et al., 2014). Furthermore, Lazarov et al. (2017) have examined the effect of ABM for cognitive-behavioral group therapy (CBGT) using a randomized controlled trial for 50 patients with SAD. They reported that the CBGT with the ABM group had greatly reduced symptoms after treatment than did the CBGT with the placebo group, and the effects were maintained at a 3-month follow-up. Since the results of the current study are suggestive of an association of J-SBST, PCN, and BDI-II with attention bias, CBT combined with ABM may be effective for individuals with chronic LBP classified as high risk with J-SBST or PCN.

This study has several limitations. First, we could not recruit an adequate number of individuals with chronic LBP; therefore, we need to expand the sample size in future studies. Second, due to the lack of a control group, we could not compare attention bias in the patients with that in healthy controls. Finally, the medication and treatment status of the participants were not effectively considered in this study, which could affect the generalization of our findings. However, this study contributes to the possibility of the development of advanced treatment for individuals with chronic LBP and is an important finding for the management of chronic pain.

## Conclusions

The findings suggest that the evaluations of pain-related psychological factors such as J-SBST or PCN or BDI-II scores are related to attention bias of individuals with chronic LBP identified by ERP and RT. In particular, the psychological scores of J-SBST and PCN related to attention bias for individuals with chronic LBP. In other words, chronic LBP patients with attention bias must assess psychosocial factors from various aspects. Furthermore, ABM may be effective in the treatment of chronic LBP older patients with attention bias, and early and middle components of ERP can also be used as one of the outcomes. Future intervention studies on treatment combined with ABM for them are necessary.

## Data Availability Statement

The raw data supporting the conclusions of this article will be made available by the authors, without undue reservation.

## Ethics Statement

The studies involving human participants were reviewed and approved by Research ethics committee of Nishikyushu University. The patients/participants provided their written informed consent to participate in this study.

## Author Contributions

TT conceived the study and participated in its design, coordination, acquisition, analysis, and interpretation of data. KM and JT conceived the study and participated in its design and interpretation of data. MM, TM, TH, AS, GH, and HT participated in data acquisition and helped draft the manuscript. All authors contributed to the article and approved the submitted version.

## Conflict of Interest

The authors declare that the research was conducted in the absence of any commercial or financial relationships that could be construed as a potential conflict of interest.
